# Implementation, enforcement, and monitoring of tobacco advertising, promotion, and sponsorship (TAPS) laws in Ethiopia: Insights from a qualitative study

**DOI:** 10.1371/journal.pone.0354842

**Published:** 2026-07-31

**Authors:** Selamawit Hirpa, Noreen Dadirai Mdege, Terefe Gelibo Argefa, Selam Abraham Kassa, Wakgari Deressa

**Affiliations:** 1 Aklilu Lemma Institute of Health Research, Addis Ababa University, Addis Ababa, Ethiopia; 2 Department of Health Sciences, University of York, York, United Kingdom; 3 Centre for Research in Health and Development, York, United Kingdom; 4 Development Gateway: An IREX Venture, Washington, District of Columbia, United States of America; 5 Department of Epidemiology and Biostatistics, School of Public Health, College of Health Sciences, Addis Ababa University, Addis Ababa, Ethiopia; Salale University, ETHIOPIA

## Abstract

**Background:**

Tobacco advertising and promotion remain major drivers of tobacco use, particularly at points of sale (PoS). Ethiopia has enacted comprehensive tobacco control laws, including bans on TAPS at PoS. However, little is known about how these laws are implemented, monitored, and enforced in practice. This study explored the facilitators and barriers to implementing, monitoring compliance with, and enforcing TAPS laws at PoS in Ethiopia.

**Methods:**

Qualitative interviews with 41 key informants were conducted between December 2022 and January 2023 across 10 purposively selected cities. Participants included policy implementers and stakeholders from government agencies, civil society organizations (CSOs), and international partners. Participants were purposively selected from national and sub-national actors involved in tobacco control, including government agencies (MoH, EFDA, Ministry of Trade, regional health and trade authorities, regulatory bodies, and research institutions) and non-governmental stakeholders (WHO Ethiopia, professional associations, and CSOs). Semi-structured interview guides were developed and administered in local languages. Interviews were audio-recorded, transcribed verbatim, translated into English, and analyzed thematically using ATLAS.ti software.

**Results:**

Key facilitators included strong tobacco control laws (Proclamation No. 1112/2019), federal-level political commitment, and the national tobacco control committee (NTCC). Barriers included the absence of EFDA structures at lower administrative levels, a lack of regionally adapted tobacco control laws, limited dedicated tobacco control departments, unclear enforcement mandates, advertisement of illicit tobacco products, not being a prioritized agenda, low awareness among non-health departments and retailers, tobacco industry interference, and, in some areas, a culture that normalizes tobacco smoking.

**Conclusions:**

Strong legislation banning 100% of TAPS and the presence of a national tobacco control committee support implementation; however, enforcement remains limited by structural, operational, and socio-cultural challenges. Enhancing compliance requires strengthening regional regulations, establishing clear institutional frameworks, raising stakeholder awareness about the tactics of the tobacco industry, and ensuring adequate allocation of human and financial resources, particularly in regions with a high prevalence of illicit tobacco products, to achieve the intended public health impact of TAPS laws in Ethiopia.

## Introduction

Tobacco use remains a leading cause of preventable morbidity and mortality worldwide, contributing to premature death, a wide range of chronic diseases, and reduced quality of life [1]. A major driver of this burden is the retail environment, which serves as a key channel for tobacco advertising and promotion, particularly in low- and middle-income countries (LMICs), where tobacco marketing remains widespread [2–4]. In 2017, Japan Tobacco International (JTI) obtained a controlling interest in Ethiopia’s National Tobacco Enterprise (NTE) after acquiring 40% of the company’s shares for US$510 million in 2016 and a further 30% stake for US$434 million in 2017, bringing its ownership to 70% [5]. Tobacco industries exploit regulatory gaps through targeted marketing, political lobbying, and other manipulative strategies, and undermine tobacco-control policies [2,3]. Among these strategies, tobacco advertising and promotion at points of sale (PoS) play a significant role, especially in shaping young people’s perceptions and behaviors. For example, exposure to tobacco advertising and promotion at PoS has been shown to significantly increase the likelihood of smoking initiation among adolescents [6,7]. Recent evidence shows that such exposure remains high among adolescents [8]. These concerns have prompted strong global and national tobacco control responses, particularly through the World Health Organization (WHO) Framework Convention on Tobacco Control (FCTC), which calls for comprehensive bans on advertising, promotion, and sponsorship (TAPS) [9,10]. Ethiopia is among the countries that signed the WHO Framework Convention on Tobacco Control (WHO FCTC) in 2004 and subsequently ratified it in 2014. Banning advertising and product displays at PoS reduces exposure to tobacco marketing, lowers youth smoking initiation, decreases overall smoking prevalence, and curbs impulse purchases among smokers [11–13]. A study showed that comprehensive tobacco advertisement, promotion, and sponsorship (TAPS) bans are associated with a 20% reduction in current smoking and a 37% reduction in the risk of smoking initiation [14].

Based on the study conducted in the year of 2016, Ethiopia has a tobacco use prevalence of about 5% among adults [15]. In 2019, the country enacted Proclamation No. 1112/2019, a comprehensive tobacco control law that aligns with the WHO FCTC and is among the strongest tobacco control measures in Africa [16,17]. This law prohibits both direct and indirect tobacco advertising and promotion, including the open display of tobacco products at PoS. Specifically, it mandates that all tobacco products be stored out of customers’ direct view. It bans the display of any advertising materials, including logos, trademarks, and promotional items, at PoS. These measures reflect a shift from earlier policies that focused primarily on the interests of the state monopoly and revenue generation [17] toward a comprehensive public health approach designed to protect the population from the harm of tobacco use.

While Ethiopia has made considerable progress in aligning its tobacco control policies with global standards, significant challenges remain in implementing, monitoring, and enforcing the policy [18]. Recent evidence shows that while non-compliance with tobacco advertising and promotion laws at outdoor PoS points-of-sale is relatively low, it remains widespread in indoor PoS [19]. However, there is a limited understanding of the challenges Ethiopia faces in complying with the ban. The purpose of this study was therefore to explore the challenges and facilitators of implementing, monitoring, and enforcing tobacco advertising and promotion bans at PoS in Ethiopia. This has been identified as a critical evidence gap by key tobacco control actors in Ethiopia [20]. The study directly advances effective tobacco control strategies in Ethiopia by providing practical insights to strengthen policy action.

## Methods

### Study setting, design, and population

This study was part of a larger project assessing compliance with TAPS laws in Ethiopia and was conducted in 10 purposively selected cities: Addis Ababa, Adama, Assosa, Bahir Dar, Dire Dawa, Gambella, Harar, Hawassa, Jigjiga, and Semera [19]. These cities were selected for their demographic and geographic diversity, higher population density, economic significance, and relatively high smoking prevalence [15].

A qualitative research design employing key informant interviews (KIIs) was used to explore the facilitators and barriers faced by actors in implementing, monitoring, and enforcing tobacco advertising and promotion bans at PoS in Ethiopia. Participants were purposively selected from key actors and partners engaged in tobacco control at both national and sub-national levels. These included government agencies such as the Ministry of Health (MoH), the Ethiopian Food and Drug Authority (EFDA), the Ministry of Trade, regional health and trade authorities, regional regulatory authorities, and research institutions, as well as non-governmental stakeholders such as WHO Ethiopia, professional associations, and civil society organizations (CSOs). Representatives from the selected agencies and organizations were selected for their involvement in tobacco control policies and programs at the national or regional level.

### Theoretical framework

To explore facilitators and barriers to implementation, compliance monitoring, and enforcement of TAPS bans, we adapted a framework based on five key explanatory factors (institutions, agendas, networks, socioeconomic factors, and ideas) previously applied in tobacco control research [21,22]. *Institutions* include both governmental bodies, from central policymakers to grassroots implementers, and non-governmental actors such as international organizations involved in tobacco control. *Agendas* refer to how tobacco is framed as a policy issue and the attention it receives. *Networks* capture the groups, organizations, and individuals with vested interests in the policy, as well as their interactions with government actors that shape policy formulation and implementation. *Socioeconomic factors* encompass the social, economic, and political context, including population behavior, demographics, and the political and economic environment where the policy is implemented. *Ideas* reflect the knowledge, beliefs, and worldviews held by policy participants, shaping policy decisions and practices.

Using this thematic framework, we systematically examined facilitators and barriers from the perspectives of national and sub-national stakeholders. This approach enabled a comprehensive understanding of the relationships among institutional structures, policy processes, and contextual factors, with a specific focus on the implementation, monitoring, and enforcement of TAPS bans at PoS in Ethiopia.

### Data collection

Semi-structured interview guides were first developed in English based on relevant literature. The guide focused on two areas. The first section briefly explored tobacco control laws in Ethiopia, including their content, formulation, the roles of key actors, and the facilitators and barriers to effective implementation. The second focused on details of TAPS laws at PoS, awareness and compliance, factors that facilitated or hindered compliance, and the role of cultural, political, and institutional contexts, with probing questions eliciting detailed responses.

The interview guides were translated into Amharic and back-translated into English to ensure accuracy. Most interviews were conducted in Amharic, while Afan Oromo and Somali were used in Adama and the Somali region, respectively.

Eleven experienced qualitative research assistants conducted the interviews. Recruitment of research assistants prioritized fluency in local languages, Amharic, and English, as well as prior experience in qualitative data collection. All research assistants participated in a three-day training course, followed by a one-day pre-testing session covering the use of the interview guides, consent procedures, recording, transcription, and translation. Pre-testing helped refine the tools and address potential challenges, with adjustments incorporated into the final interview guide. All interviewers adhered to standardized procedures throughout the data collection process to maintain consistency and data quality.

Key stakeholders and policy implementers from government offices and NGOs/CSOs were contacted in person or by phone and provided with study information. The research team also used this encounter to schedule interviews with those who were willing to participate. They were informed about the study’s context and how the data were to be collected and used. All interviews were conducted in person in the key informant’s office or a private place of their preference. On average, the interviews lasted 40–90 minutes and were digitally recorded with prior consent. Following each interview, the interviewers wrote field notes summarizing key points, which were discussed during debriefings. Data collection was conducted between December 9, 2022, and January 5, 2023, with the research team visiting the study sites to support fieldwork. All selected institutions, along with their representatives, participated in the study.

### Data analysis

All interviews were transcribed verbatim to word files, anonymized, and translated into English. The transcripts were independently reviewed and coded by three experienced researchers. A combination of deductive and emergent coding was applied, with transcripts read multiple times to ensure familiarity with the content. The research team developed an initial set of codes, which was iteratively refined as additional transcripts were reviewed, leading to a final codebook. The team discussed and resolved coding discrepancies to reach consensus. Codes were then grouped into themes and subthemes to create a thematic matrix, and an iterative analysis was conducted to identify key facilitators and barriers to the implementation, monitoring, and enforcement of TAPS bans at PoS. All transcripts and codes were uploaded into ATLAS.ti version 8 software for thematic analysis. The analysis was guided by the overarching research objectives, which involved identifying and coding relevant text segments. Selected verbatim quotes were presented using anonymous names and codes to illustrate key findings.

In this study, PoS refers to any location where cigarettes and other tobacco products are advertised, displayed, promoted, or sold. Implementation describes the process of implementing tobacco advertising and promotion bans at PoS, including how the laws are introduced, communicated, and adopted in retail settings. Monitoring involves assessing compliance with these bans through inspections or other oversight mechanisms, while enforcement covers actions taken when violations occur, such as sanctions or corrective measures. Examining these dimensions through the lens of institutions, agendas, networks, socioeconomic factors, and ideas provides a comprehensive understanding of the facilitators and barriers affecting the effectiveness of TAPS laws in Ethiopia.

### Ethics statement

Ethics approval for the study was obtained from the Institutional Review Board of the Ethiopian Public Health Association (EPHA) (EPHA/OG/201/22; November 18, 2022). Interview guides did not include names, addresses, or any other identifying information, ensuring anonymity, privacy, and data confidentiality. Each participant provided written consent for the interview and agreed to an audio recording.

## Results

### Characteristics of participants

A total of 41 key informants participated in the interviews (Table 1). Most were men (90.2%) and represented federal/national (29.3%), regional (56.1%), EFDA (19.5%), and health sector (43.9%) institutions. In Addis Ababa, 14 interviews were conducted with participants from EFDA, MoH, Addis Ababa, and Oromia Health Bureaus, WHO, EPHI, CTFK, CSOs, and the Ministry of Trade. Health bureaus, regulatory offices, and trade authorities mainly represent participants from the nine regional cities and district offices.

**Table 1:** Characteristics of participants.

Characteristics	Frequency, n	Percentage, %
**Gender**		
Male	37	90.2
Female	4	9.8
**Organizational level**		
Federal/National	12	29.3
Regional	23	56.1
Zonal/District	6	14.6
**Types of organization**		
MoH/Regional/Zonal/District Health Bureaus	19	46.3
National/Regional/Zonal/District Trade Offices	8	19.5
EFDA/Regional Regulatory Bodies	8	19.5
WHO/NGOs/CSOs	5	12.2
Ethiopian Public Health Institute	1	2.4
**City’s level**		
Addis Ababa	14	34.2
Regional cities	27	65.8
**Total, n**	**41**	**100.0**

### Facilitators for implementation, monitoring, and enforcement of TAPS laws at the POS in Ethiopia

Participants noted that the implementation, monitoring, and enforcement of TAPS laws at points of sale (POS) in Ethiopia were facilitated by the presence of tobacco control laws and directives, strong political leadership and commitment, active multisectoral collaboration, and negative societal attitudes toward tobacco use in most areas.

#### Institution.

**Tobacco control laws and directives.** Nearly all respondents noted that Ethiopia has strong tobacco control laws (Proclamation 1112/2019), including the Tobacco Advertisement, Promotion, and Sponsorship (TAPS) law at the point of sale (PoS). The approved tobacco control law is considered comprehensive and in line with the WHO Framework Convention on Tobacco Control (WHO FCTC). Unlike the 2015 directive, the current tobacco control law [Proclamation 1112/2019] is more comprehensive and includes provisions on penalties for noncompliance. The improved scope and strength of the revised law were regarded as facilitating better tobacco monitoring and enforcement, as well as additional community-based awareness-raising activities. According to respondents, this has led to progressive improvement in the understanding of tobacco control laws, particularly in urban areas.

A participant from Addis Ababa reported that:

*The law is strong, which you can take as an opportunity. The other thing is the professionals’ [experts working in the tobacco control area] commitment, which is a plus.*

*[Tobacco control expert, Addis Ababa]*

#### Agenda.

**Political leadership and commitment.** Most participants agreed that there is a strong commitment by the political leadership to tobacco control, in general, as reflected in TAPS laws. This is evidenced by the approval of tobacco control laws and the increase in tobacco tax.

An interviewee stated that:

*……. the leadership is committed; it has approved the proclamation [1112/2019] …… if they [political leaders] were not committed, the proclamation would not have been approved and…*

*[Inspector at the regional trade office]*

#### Networks.

It was noted that a tobacco control committee exists at the national level, with representatives from various health and non-health departments. The same is also true for some regions. The monitoring and enforcement of TAPS laws require multi-sectoral collaboration, and these networks were essential for implementing the activities.

A participant indicated that:

*The police, as well as other law enforcement agencies and business offices, are among the stakeholders involved in this [Tobacco control committee] … Each region has its own steering committee, which meets regularly with stakeholders to evaluate the work. At the regional level, they establish their own tobacco control committees and hold several meetings…*

*[Tobacco control expert at the federal level].*

#### Socioeconomic factors.

**Culture, societal norms, and religion.** Perception towards tobacco use varies across regions due to the range of cultures and differences in social norms. In eastern parts of the country, tobacco use is part of the culture and regarded as part of the daily routine. However, in most parts of the country, people do not have a positive attitude towards tobacco use. This provides a conducive environment to implement TAPS laws and leverages regulatory interventions to ensure compliance.

A participant from one of the regional health bureaus reported that:

*In some places, sitting and smoking in public is seen as a shameful act……. Our society does not encourage smoking.*

*[Regulatory office staff from a regional health bureau]*

### Barriers to the implementation, monitoring, and enforcement at the PoS in Ethiopia

Study participants highlighted several barriers to implementing, monitoring, and enforcing TAPS (Tobacco Advertising, Promotion, and Sponsorship) laws at points of sale (POS) in Ethiopia. These included the lack of structured systems at the lower district level, the absence of a dedicated department focused solely on tobacco control, and unclear organizational mandates. High workloads were also identified as a significant barrier. Additionally, participants noted that TAPS laws are not consistently prioritized on the policy agenda, with limited political leadership and commitment in certain areas. Other challenges included insufficient intersectoral collaboration, the cultural acceptance of tobacco use in some regions, low awareness of TAPS laws, tobacco industry interference, and the promotion activities of illicit tobacco products.

#### Institution.

**Lack of structure at the lower district level.** In all regions, the EFDA lacked a lower-level structure to monitor and enforce TAPS laws. It was noted that, unlike smoke-free laws, there is no EFDA structure for monitoring and enforcing the TAPS law at the district level, including in the capital city (Addis Ababa).

An interviewee said that:

*… our structure has a gap…... The Ministry of Health and the Ministry of Education each have their own structures up to the district level. For example, there is an education office at the district level. But for food and drug authorities, there is no district-level structure across all regions. Our authority office operates only at the regional level.*

*[Tobacco control expert from EFDA]*

A second interviewee stated that:

*We don’t have a structure [to monitor and enforce the TAPS law]. It is highly problematic, as the structure is not available at the district level… It only exists at the regional level. ……. There is no structure, office, or monitoring at the district level……This is very difficult….*

*[A study participant from the Regional Health Office]*

**Lack of a department uniquely working on tobacco control.** In general, the health sector was perceived by most as a leader for tobacco control activities with clear institutional roles. However, the administrative structures and institutional roles of the remaining stakeholders in tobacco control were found to be unclear and inconsistent across regions. Although some participants reported the existence of a task force, most agreed that there is no department or standalone tobacco control case team. This was mentioned by regional experts, including federal-level staff. Tasks related to tobacco control are added to other departments, such as drug abuse prevention case teams. Similarly, a responsible body was reportedly not assigned to oversee specific tasks related to compliance with TAPS law and the enforcement of tobacco control laws in general. Alternatively, individuals tasked with monitoring and law enforcement are assigned additional responsibilities that may not directly relate to tobacco control.

An interviewee stated that:

*The regulatory body should be independent……. It would be preferable for the regulatory body to be separated from the health bureau. Our work is challenging because the two entities are combined, causing interference. We struggle to focus on enforcing tobacco control laws.*

*[Participant from the Regional Health Bureau]*

A second interviewee stated that:

*…… There is no single body responsible for a single task, such as controlling and monitoring, inspecting, implementing, and enforcing the law. So, I do not think responsibilities are assigned separately to different bodies. We in the health sector try to address community awareness issues, though we have many responsibilities. I doubt if the laws [Tobacco control laws] implementation was well studied because it was just approved, but a clear direction was not given for the implementation”*.

*[Non-communicable disease expert; Regional Health Bureau]*

**Lack of clarity on organizational mandate.** Participants noted a lack of clarity regarding the mandates of different stakeholders for monitoring and enforcing TAPS laws. For instance, the Ministry of Trade is mandated to provide licenses and inspect regular shops. However, there is a pervasive lack of clarity on the position of the trade office and other stakeholders on tobacco control. This has resulted from EFDA’s widespread recognition as a leading organization in tobacco control. This causes a significant gap in the implementation, monitoring, and enforcement of TAPS laws.

A participant pointed out that:

*The Ministry of Trade issues licenses to retail stores… and the health department doesn’t issue [a license], so how can the health department have a mandate [for TAPS law monitoring and enforcement]? …. [Tobacco control expert from Addis Ababa]*

A second interviewee said that:

*……. It is not our role to stop advertising; this responsibility falls to the Office of Trade. As a monitoring institution under the health sector, our focus includes hotels, parks, restaurants, cafeterias, youth centers, and both public and private health centers.*

*[An interviewee, FMHACA Regional Bureau]*

**High workload.** Except in a few regions, most have assigned the regulation of tobacco law compliance to departments with pre-existing responsibilities. This has led to competing priorities and an increased workload for staff. Considering the need for frequent on-site visits and strong stakeholder engagement to monitor implementation, the increased workload and time constraints impede compliance assurance efforts.

A participant mentioned that:

*Three people in this big city are not enough to work on monitoring and law enforcement of the TAPS law [An interviewee from the region].*

A second interviewee mentioned that:

*There is a huge problem with human resources… if you go to each district, you’ll find that only one person is in charge of everything. This individual is the head of public and private health institutions, health and health-related issues, tobacco control, traditional medicine, and drug control. So, he will burn out [due to the workload]. [A study participant, Regional Regulatory body]*

**Tobacco industry interference.** Participants emphasized that the tobacco industry may interfere, directly or indirectly, with the implementation of the TAPS laws. The industry provided tobacco promotional items to the retailers, discounted the price of cigarette packets with graphic health warnings, and increased the market circulation of illicit tobacco products. For instance, the tobacco industry worked with the trade office to curb illegal tobacco products in one of Addis Ababa’s sub-cities. Once the illicit tobacco products were confiscated from the point of sale, they would be given to the tobacco industry.

An interviewee stated that:

*……. Right now, the tobacco industry is collaborating with us. First, when we confiscated the illicit tobacco products at the point of sale, we returned it [illicit tobacco product] back to the tobacco industry.*

*[Business management and control expert; district trade office in Addis Ababa]*

A second participant reported that:

*When the tobacco control law on graphic health warning was introduced at first time, the tobacco industry intentionally said that, a two-year tobacco product is already produced, so either you pay us the costs or we will consider a graphic warning after two years……so it was allowed the cigarette packet without the graphic health warning to stay in the market for two years and at the same time cigarette packets with graphic health warning was introduced to the market…….then the tobacco industry reduced the price of cigarettes packets with the graphic health warning to 35 Birr and raised the price to 85 birr for cigarette packet without a graphical warning. You cannot fix this by monitoring it; it is a free market. [An interviewee from Addis Ababa].*

**Regions lack implementation guidelines/directives.** More than half of the participants reported that there was a lack of implementation of guidelines and directives for TAPS laws, which are vital to enforce the law and monitor compliance, as a barrier to the implementation, monitoring, and enforcement. A participant mentioned that:

*… the tobacco control proclamation has not been adopted in all regions. So far, it has only been adopted in one region, Dire Dawa. [A participant from a federal office]*

*A second interviewee stated that:*

*In our region, we have not yet adopted Proclamation 1112/2019. [Regulatory body from the Regional Health Bureau]*

#### Agenda.

**Political leadership and commitment.** Political leadership and commitment are essential for compliance, monitoring, and enforcement of TAPS law. However, in areas like the eastern part of Ethiopia, participants reported a lack of commitment that was partly explained by the fact that tobacco use is part of the social norm.

An interviewee said that:

*There is no political commitment for TAPS law implementation if you come to our region……if higher political leaders were committed, there would have been a lot of change [TAPS law compliance, monitoring, and enforcement].*

[An interviewee from the region]

**Tobacco control is not set as a priority agenda.** Some participants reported that tobacco is not regarded as a priority on the agenda. Offices in a few regions have competing agendas and prioritize other issues that appear more urgent. Even when tobacco control is not the agenda, more attention is given to smoke-free laws than to TAPS laws. A participant from a regional trade office reported that:

*“ ….. This [TAPS laws monitoring and enforcement] is not considered a priority. There are other competing agendas, such as regulating the cost of living and other pressing matters. These issues make stakeholders busy and create a gap in monitoring and enforcement of the law [tobacco control laws].*

*[Inspector from the Regional Trade Office]*

Another participant pointed out that:

*As a country and as a city, many of us are working on smoke-free laws; however, control over advertising is lagging. Our control is to prevent people from smoking in places where smoking is prohibited. Not much is being done on TAPS.*

[A participant from the Regional Regulatory Office]

#### Networks.

**Limited intersectoral collaboration for tobacco control.** The TAPS law’s monitoring and enforcement at the point of sale require multisectoral collaboration, but establishing this collaboration poses a challenge that calls for a strong coordinating body. Stakeholders are not equally committed to enforcing the tobacco control laws. The relationship between the Ministry of Trade, which issues business licenses to tobacco retailers, the EFDA, and law enforcement offices is limited. For instance, inspectors from the Ministry of Trade are not as engaged in supervising the enforcement of TAPS laws. At times, law enforcement officers are reluctant to respond to requests for collaboration from other stakeholders, especially at the regional level. Participants recommended that once a strong collaboration between stakeholders is established, the existing structure of the Ministry of Trade and Customs Office is robust enough to monitor compliance with TAPS laws. It was discussed that regular meetings with all concerned stakeholders are needed, and that a strong coordinating institution is required.

A participant mentioned that:

*Of course, yes, there is a significant lack of coordination. The food and drug authority, trade office, and health office each have a specific role; however, all offices assume that tobacco control is the responsibility of the food and drug authority. On the other hand, the FDA considers it as the responsibility of the trade office…. There is a lack of coordination between the stakeholders.*

*[An inspector in the Ministry of Trade Office]*

#### Socioeconomic factors.

**Culture and societal norms.** In some regions, cigarette smoking is part of various cultural traditions. In such settings, enforcing TAPS laws and ensuring subsequent compliance are challenging. In areas where smoking is part of everyday life, a considerable proportion of people use tobacco products, and points of sale (POS) are an integral part of this environment. The fact that it is part of cultural traditions challenges the implementation of TAPS laws. However, tobacco is widely accepted only in a limited part of the country.

One participant reported that:

*In this region, there are social challenges to tobacco control; smoking is considered part of the culture. So, there is high acceptance [for cigarette smoking] … [people will say] Why are you telling us not to sell a single cigarette while the government is receiving funds and opening a factory like this? ………In fact, there is a situation in which ‘Gaya’ [Traditional tobacco] is considered a form of wealth and given as a gift to relatives or community members, reflecting a cultural tradition.*

*[A study participant from the region]*

**Tobacco as a source of income.** Participants noted that some individuals derive their livelihoods from the tobacco trade. These people and businesses have competing interests with tobacco control efforts and try to undermine compliance with the TAPS laws.

An interviewee stated that:

*Nowadays, people want money, and they will do anything to earn more. For example, the farmers I mentioned earlier, if given a lot of money to grow tobacco, may stop growing wheat and switch to tobacco. Similarly, illicit tobacco product sales may increase, or people may ignore TAPS law compliance if it is a source of income [Regulatory body from region].*

**Illicit tobacco products.** Illicit tobacco products were regarded as obstacles to compliance with TAPS laws by the participants. There are different brands of illicit tobacco products in the market, accompanied by other forms of advertisement aimed at increasing market share. Participants from eastern Ethiopia frequently reported this. The frequently reported approach is to post their brands on durable plastic bags used to carry household consumables. Furthermore, the presence of illicit tobacco products was mentioned as a barrier to the monitoring and enforcement of TAPS laws.

An interviewee stated that:

*… the most advertised cigarettes are illicit in the country, and the reason why they need to advertise is to get more consumers, 70% to 90% of cigarettes in the city are not registered [are illicit] ……. There is a high concentration of illegal tobacco products, including indirect tobacco advertisements using umbrellas, plastic bags, T-shirts, and key-holders.*

*[Regulatory body from the eastern part of Ethiopia*]

A second interviewee stated that:

*We are at the border where 100 types of cigarettes enter the country daily, each with its own special advertisements. If you go to Jigjiga city today, you will see something you could never imagine: big 3D posters, large cigarette banners printed on bags sold everywhere, and T-shirts with cigarette advertisements worn by poor people… Most of the advertised cigarettes are unregistered… They need to promote because inflation is high. They want to get more consumers…*

*[Regulatory body from region].*

**Lack of resources.** The other challenge is that there are numerous shops, which require resources, including human resources and budget. In addition to financial limitations, several regulatory bodies are also preoccupied with other responsibilities. Regions should adopt strong tobacco control laws (Proclamation 1112/2019) for their own, and a specific implementation guideline is necessary. If this is the case, the budget will also be allocated for the tobacco control implementation activity. This makes monitoring and law enforcement activity transparent and easy.

A participant stated that:

*We can say 100% that there is no resource to monitor and enforce TAPS laws. Every activity and every task requires funds… This [TAPS law monitoring and enforcement] is extensive work… Tobacco smoking has been in the community for a long time… so it is difficult to do this work without funds.*

*[An interviewee from the Regional Health Bureau]*

#### Ideas.

**Knowledge and perception.** Effective monitoring and enforcement of TAPS laws needs collaboration between a range of institutions. For this reason, all actors need to have adequate knowledge of the law, including the implementation guidelines and directives. Participants frequently noted a knowledge gap among staff across different government offices regarding tobacco control laws in general and TAPS laws, which they viewed as hindering their effective implementation. It undermined accountability, collaboration among stakeholders, and, more importantly, the assignment and taking of responsibility in tobacco control-related efforts. Among the government offices involved in this study, experts at various levels frequently reported a lack of awareness of TAPS laws. Apart from experts at the regulatory offices, shop owners, and the public were also reported to have inadequate knowledge of the laws.

An interviewee pointed out that:

*The community, especially retailers, doesn’t understand that plastic shopping bags displaying cigarette brands are designed for cigarette advertising.*

*[Staff at one of the regional offices]*

A second participant stated that:

Staff from the regional ministry of trade office admitted that.

*I don’t have any information [about TAPs law] ……… I mean, at the very least, everyone should be aware, because I believe I am not as aware as I should be…”*

*[Inspector from the regional Ministry of Trade office]*

#### Network.

Lead organizations in tobacco control in the indicated regions reported having limited partners (governmental and other organizations) with whom they can network. This posed a challenge for TAPS law implementation and enforcement, as the work is extensive given the many points of sale, but staffing for supervision is limited.

A study participant said that:

*No other government or charitable organization is working on this [TAPS law monitoring and law enforcement] in our region, so implementing it will be challenging.*

*[Regulatory body from the regional office]*

## Discussion

This study explored the facilitators and barriers to implementing the TAPS law in Ethiopia, using a framework comprising five main explanatory factors: institutions, agendas, networks, socioeconomic factors, and ideas. The key facilitators of TAPS law implementation identified by participants included strong, comprehensive tobacco control laws, strong national-level political commitment, the involvement of multiple stakeholders in tobacco control activities, and prevailing cultural and social norms that discourage tobacco use. Conversely, several barriers hindered the effective implementation of TAPS laws. These included the absence of structured systems working on TAPS laws at the lower administrative levels, the lack of a dedicated department responsible for tobacco control, unclear organizational mandates, the absence of regional implementation guidelines, limited prioritization of TAPS within policy agendas, illicit tobacco trade, cultural practices that normalize tobacco use, tobacco as a source of livelihood, and interference from the tobacco industry.

The lack of structure at lower administrative levels and the absence of a dedicated tobacco control department are identified as significant barriers to TAPS law monitoring and enforcement at points of sale. Participants frequently compared TAPS law implementation with smoke-free laws, noting that the EFDA had a clear structure at the lower level to oversee smoke-free law enforcement. In contrast, the Ministry of Trade is responsible for issuing licenses to retail outlets and enforcing TAPS laws. However, participants reported that the mandate for enforcing TAPS laws is unclear, and many are unaware of their existence. In agreement, a study conducted in Kenya reported uncertainty on the mandate and responsibility for enforcing the tobacco control laws [23]. Limited resources were also identified as a key challenge. This is despite WHO recommendations that parties designate a competent, independent authority to monitor and enforce the TAPS laws, entrusting it with the necessary powers and resources [24]. Similar to our findings, studies conducted in Gambia and Lebanon also mentioned that the Lack of awareness is a barrier to TAPS laws monitoring and enforcement [25,26].

Participants emphasized that the strong tobacco control law, *Proclamation 1112/2019* [4], which is a leading facilitator of TAPS law implementation, monitoring, and enforcement, mandates a 100% ban on TAPS and serves as a critical facilitator of its implementation, monitoring, and enforcement. The enactment of this law was viewed as an indication of strong political leadership and commitment. In line with this study, prior evidence shows that tobacco control policies are effective in reducing tobacco demand and increasing the implementation of measures across several policy domains when implemented in accordance with their guidelines [27–29].

At the subnational level, regional administration offices are expected to regulate the implementation of the TAPS law. To ensure compliance, regions should adapt their tobacco control regulations to the federal proclamation. However, at the time of the study, most regions relied solely on the general federal tobacco control proclamation. The lack of region-specific guidelines and directives was reported as a major barrier to effective implementation. Moreover, TAPS law enforcement was not prioritized at either the national or regional levels compared to smoke-free laws. Consistent with the current study, previous research has highlighted the importance of implementing TAPS laws at the subnational level, particularly in large countries like Ethiopia, and has emphasized the need for active subnational tobacco control committees. Moreover, Political commitment and inter-organizational networks also play a critical role in enhancing tobacco control implementation, monitoring, and enforcement [30,31].

Participants further explained that in many Ethiopian cultures and religious contexts, tobacco smoking is socially unacceptable, which facilitates the implementation of TAPS laws. In such settings, point-of-sale (PoS) owners tend to comply readily with TAPS regulations when instructed by law enforcement authorities. However, this facilitating context is not uniform across the country. In some communities, tobacco use remains culturally embedded, creating challenges for the effective implementation of TAPS laws. This is also documented in previous studies, where socio-cultural differences affected the TAPS law implementation, monitoring, and enforcement [19,32].

The study revealed that the advertisement of illicit tobacco products at points of sale (PoS) remains a significant obstacle to the effective implementation, monitoring, and enforcement of TAPS laws. Participants reported instances such as the use of plastic bags displaying tobacco brand advertisements at retail outlets. In addition, it was noted that the tobacco industry’s involvement in government-led illicit tobacco control activities contradicts Article 5.3 of the WHO Framework Convention on Tobacco Control (FCTC), which requires countries to protect public health policies from the tobacco industry’s vested interests [33]. Furthermore, participants indicated that the tobacco industry itself is involved in the sale of illicit tobacco products and frequently fails to comply with TAPS regulations. Similar patterns of industry interference as barriers to TAPS law implementation have been documented in several other countries [34–35].

### Strengths and limitations of this study

This is the first qualitative study to explore the facilitators and barriers to implementing, enforcing, and monitoring TAPS laws in Ethiopia. It draws on rich data from key informant interviews conducted across 10 major cities, involving participants from both health and non-health departments. The study provides valuable contextual evidence that can inform future policy design, enforcement strategies, and capacity-building initiatives related to tobacco control.

However, the study relied solely on key informant interviews, and most participants served as focal people for tobacco control. This may have introduced reporting bias, as participants could under- or overreport the extent of TAPS implementation, monitoring, and enforcement. Furthermore, including retail point-of-sale representatives could have provided more insights into the implementation of TAPS laws at the ground level.

## Conclusions

In conclusion, strong national legislation, federal political commitment, the national tobacco control committee, and multisectoral collaboration facilitate enforcement in Ethiopia. However, several challenges hinder compliance, including the absence of EFDA structures at lower levels, limited regional regulations, unclear mandates, low awareness among non-health departments, and interference from the tobacco industry. Socio-cultural factors both support and impede tobacco control, depending on local norms. Strengthening enforcement requires leveraging these facilitators while addressing structural, cultural, and operational barriers through clear institutional frameworks, regional regulations, coordinated networks, creating awareness about the tactics of the tobacco industry for the public and stakeholders, and adequate financial and human resources, with particular attention to regions facing high levels of illicit tobacco products.

## Abbreviations

CSOS: Civil society organizations
CTFK: Campaigns for Tobacco Free Kids
EFDA: Ethiopian Food and Drug Authority
EPHA: Ethiopian Public Health Association
EPHI: Ethiopian Public Health Institute
FCTC: Framework Convention on Tobacco Control
KIIs: Key informant interviews
LMICs: Low- and middle-income countries
MOH: Ministry of Health
NGOs: Non-governmental organizations
NTCC: National Tobacco Control Committee
PoS: Points-of-sale
TAPS: Tobacco advertising, promotion, and sponsorship
WHO: World Health Organization
